# Estimated Glomerular Filtration Rate, Nutritional Factors, and Their Relationships With Homocysteine in Community‐Dwelling Older Adults

**DOI:** 10.1155/jnme/7471621

**Published:** 2026-06-15

**Authors:** Hsiu-Lan Li, Yao-Mei Chuang, Po-Jung Huang, Po-Sheng Chang

**Affiliations:** ^1^ Department of Nursing, En Chu Kong Hospital, New Taipei City, Taiwan, eck.org.tw; ^2^ Department of Health and Leisure Management, Yuanpei University of Medical Technology, Hsinchu City, Taiwan, ypu.edu.tw

**Keywords:** folic acid, homocysteine, older adults, renal function, vitamin B_12_

## Abstract

Age‐related renal function decline may contribute to other physiological alterations. This study aimed to examine the associations of kidney function, assessed by estimated glomerular filtration rate (eGFR), and nutritional factors with homocysteine levels in community‐dwelling older adults. This cross‐sectional study enrolled participants aged ≥ 65 years. Demographic characteristics were collected using questionnaires. Biochemical parameters, including homocysteine, folic acid, vitamin B_12_, and metabolic disorder indicators, were measured, and eGFR was calculated and categorized into three groups. Homocysteine concentrations were significantly higher in the low and moderate eGFR groups compared with the high eGFR group (median: 16.9 vs. 11.9 vs. 9.3 μM, *p* < 0.01). Regression analyses indicated that each 1‐unit decrease in eGFR was associated with higher homocysteine (*β* = −0.13, *p* < 0.01). Conversely, higher folic acid (*β* = −0.29, *p* < 0.01) and vitamin B_12_ (*β* = −0.003, *p* < 0.01) levels were significantly associated with lower homocysteine concentrations. These associations remained significant after mutual adjustment, indicating distinct effects of renal function and nutritional factors on homocysteine levels. Moreover, homocysteine levels were positively correlated with waist circumference (*r* = 0.38, *p* < 0.01) and triglycerides (*r* = 0.18, *p* < 0.05) and inversely correlated with high‐density lipoprotein cholesterol (*r* = −0.27, *p* < 0.01). The results suggest that renal function and nutritional status are independently associated with homocysteine levels and may contribute to metabolic risk profiles in older adults.

## 1. Introduction

Senescence can lead to a decline in various physiological functions, including renal function. During this process, the number of nephrons naturally decreases, prompting the remaining nephrons to undergo compensatory hypertrophy, which may ultimately cause podocyte injury and loss [1, 2]. Moreover, comorbid conditions such as hypertension, diabetes, and obesity can exacerbate this process, contributing to a progressive decline in glomerular filtration rate [3]. As chronic kidney disease (CKD) is progressive and irreversible because nephrons have limited capacity for repair, greater attention should be paid to renal health to prevent further complications [4]. Early manifestations of declining renal function may include excessive fatigue, sleep disturbances, and musculoskeletal discomforts such as bone or joint pain [5]. However, these symptoms are rarely assessed systematically in relation to renal function, and their potential association with renal decline is often overlooked, leading to underestimation of renal impairment in clinical practice [6]. Evidence from the Korean Frailty and Aging Cohort Study indicates that kidney impairment is associated with age‐related cognitive decline in older adults, even in cases of mild dysfunction [7]. This highlights the importance of monitoring other physiological parameters in seniors experiencing progressive kidney deterioration. Slowing or delaying further physiological decline may help reduce the severity of complications. Therefore, even a modest reduction in kidney function warrants clinical attention and should not be underestimated.

Homocysteine in the circulation is primarily cleared by the kidneys, and elevated plasma homocysteine concentrations have been observed in middle‐aged individuals with CKD, as demonstrated in a large cross‐sectional cohort study [8]. Biochemically, homocysteine is a sulfur‐containing amino acid whose metabolism depends on B vitamins, particularly folic acid and vitamin B_12_, which serve as essential cofactors for its remethylation to methionine. Adequate B vitamin status facilitates this conversion and has been associated with reduced risks of cardiovascular disease and all‐cause mortality [9]. In a recent commentary, Hirata noted that findings regarding the impact of hyperhomocysteinemia on disease risk remain conflicting across studies [10]. From another perspective, one may consider that declining renal function may impair homocysteine metabolism, potentially leading to elevated circulating homocysteine levels, which have been associated with increased cardiovascular risk. Since kidney aging is an inevitable biological process [11], identifying factors involved in the regulation of plasma homocysteine levels is particularly important. Although previous studies have reported associations between elevated homocysteine levels and CKD in middle‐aged and elderly populations [12], most of these studies have examined homocysteine primarily as a predictor or biomarker of CKD in general adult populations, rather than focusing specifically on how varying levels of renal function relate to homocysteine concentrations within older individuals. In addition, renal function and nutritional factors involved in homocysteine metabolism, such as folic acid and vitamin B_12_, have often been examined separately, and their concurrent roles to homocysteine levels in older adults have not been fully clarified. Therefore, the present study aims to examine the association between kidney function, assessed by estimated glomerular filtration rate (eGFR), and homocysteine concentrations in community‐dwelling older adults, while simultaneously evaluating whether nutritional factors are involved in homocysteine metabolism.

## 2. Methods

### 2.1. Participants

This cross‐sectional study recruited independently living older adults aged over 65 years from community activity centers in Taiwan, where regular social and health‐related activities are held. The exclusion criteria were as follows: (1) presence of critical illnesses, such as cancer, severe heart or lung disease, liver cirrhosis, or end‐stage CKD; (2) physical disability; and (3) cognitive impairment. The study protocol was reviewed and approved by the Institutional Review Board of En Chu Kong Hospital, Taiwan (IRB No. ECKIRB1130902), and all procedures involving human participants adhered to the ethical standards of the Declaration of Helsinki. Informed written consent was obtained from all individuals prior to participation.

### 2.2. Characteristics and Anthropometric Assessments

Participant characteristics, including age, gender, and self‐reported medical history, were obtained through structured questionnaires administered to all participants. Blood pressure was measured using an automated digital electronic sphygmomanometer. Height and weight were measured using a stadiometer and calibrated scale, respectively, and body mass index (BMI, kg/m^2^) was subsequently calculated. Waist, hip, and calf circumferences were measured using a nonstretchable measuring tape, and the waist‐to‐hip ratio (WHR) was subsequently calculated.

### 2.3. Blood Sample Collection and Biochemical Analyses

Participants were instructed to fast for at least 12 h prior to blood sample collection. Fasting venous blood samples were collected using vacutainer tubes containing K_2_‐EDTA (for glycated hemoglobin [HbA1_C_] measurement), no anticoagulant (for serum analyses), and sodium fluoride with potassium oxalate (for glucose measurement). Serum was separated by centrifugation at 3000 rpm for 15 min at 4°C, and the supernatant was collected for subsequent biochemical analyses. Biochemical parameters, including fasting blood glucose (FBG), blood urea nitrogen (BUN), creatinine, aspartate aminotransferase (AST), alanine aminotransferase (ALT), total cholesterol (TC), high‐density lipoprotein cholesterol (HDL‐C), low‐density lipoprotein cholesterol (LDL‐C), triglycerides (TGs), and uric acid, were measured using a fully automated high‐throughput photometric analyzer (cobas c702 module, Roche Diagnostics). The Premier Hb9210 employs a patented boronate affinity high‐performance liquid chromatography method to measure HbA1_C_. Homocysteine levels were determined using an automated immunoassay analyzer (Alinity i, Abbott Diagnostics). Hyperhomocysteinemia was defined based on sex‐specific clinical reference values as a homocysteine level > 16.20 μM in males or > 13.56 μM in females. Serum folic acid and vitamin B_12_ concentrations were measured on the cobas e801 and cobas e602 modules, respectively, using fully automated immunoassay systems (Roche Diagnostics).

### 2.4. Estimation of eGFR and Group Classification

Renal function was assessed using the Chronic Kidney Disease Epidemiology Collaboration (CKD‐EPI) equation, which has been shown to classify individuals more accurately by mortality and end‐stage renal disease risk compared with the Modification of Diet in Renal Disease (MDRD) formula in older populations [3, 13]. The CKD‐EPI formula is eGFR (ml/min/1.73 m^2^) = 142 × min (Scr/K, 1)^α^ × max (Scr/K, 1)^−1.200^ × 0.9938^age (years)^ × 1.012 (if female). Scr = serum creatinine concentration (mg/dL); *K* = 0.9 for males, 0.7 for females; α = −0.302 for males, −0.241 for females; min = the smaller value between Scr/K and 1; max = the larger value between Scr/K and 1 [14]. In the present study, eGFR was used solely as an indicator of renal function status and not for clinical diagnosis of kidney disease. Participants were classified into three groups based on eGFR values: high eGFR group—eGFR ≥ 90 mL/min/1.73 m^2^; moderate eGFR group—60 mL/min/1.73 m^2^ ≤ eGFR < 90 mL/min/1.73 m^2^; and low eGFR group—eGFR < 60 mL/min/1.73 m^2^. These categories correspond approximately to CKD stages G1, G2, and ≥ G3, although eGFR was used in this study as an indicator of renal function rather than for clinical staging.

### 2.5. Statistical Analyses

All statistical analyses were conducted using SigmaPlot (Version 12.0). Descriptive statistics for continuous variables were expressed as mean ± standard deviation (median). Categorical variables were summarized as frequencies and percentages. The Shapiro–Wilk test was used to assess the normality of data distribution. One‐way analysis of variance (ANOVA) or the Kruskal–Wallis test was used to compare continuous variables among the low, moderate, and high eGFR groups. The chi‐square test or Fisher’s exact test was used for comparisons of categorical variables. Multiple linear regression analysis was performed to assess the associations between renal function (eGFR) and nutritional factors (folic acid and vitamin B_12_) with homocysteine levels. These variables were simultaneously included in the regression models to evaluate their independent associations. Homocysteine was treated as the dependent variable, and eGFR was considered the primary independent variable in the regression analyses. Covariates were selected based on clinical relevance and prior evidence rather than solely on statistical significance in univariate analyses. Multiple logistic regression analysis was used to evaluate the associations of renal function indicators and nutritional factors with the risk of hyperhomocysteinemia. For comparisons between participants with and without hyperhomocysteinemia, Student′s *t*‐test or the Mann–Whitney *U* test was used for continuous variables. Spearman’s rank‐order correlation coefficient was used to examine correlations between homocysteine levels and metabolic syndrome indicators. A *p* value of less than 0.05 was considered statistically significant.

## 3. Results

### 3.1. The Characteristics and Biochemical Data of the Participants According to the Levels of eGFR

A total of 120 elderly participants were enrolled in the present study, with a mean age of 75.2 years and 28.3% being male. Table 1 shows the characteristics of the elderly participants stratified by renal function based on eGFR values. Participants in the low and moderate eGFR groups had significantly higher median values of age (81.0 vs. 76.5 vs. 72.0 years, *p* < 0.01) and WHR (0.95 vs. 0.92 vs. 0.88, *p* = 0.01) compared to those in the high eGFR group. In addition, the proportion of participants with heart disease was slightly lower in the high eGFR group compared to the moderate and low eGFR groups (*p* = 0.06).

**TABLE 1:** Characteristics of the participants according to the level of eGFR.

	**High eGFR (*N* = 56)**	**Moderate eGFR (*N* = 54)**	**Low eGFR (*N* = 10)**	**p** **value**

Age (years)	72.3 ± 4.2 (72.0)^1,a^	76.9 ± 5.0 (76.5)^b^	82.2 ± 6.9 (81.0)^c^	**<** **0.01**
Male (*n*, %)	15 (26.8%)	15 (27.8%)	4 (40.0%)	0.69
SBP (mmHg)	140.3 ± 20.6 (138.0)	137.9 ± 16.8 (134.0)	148.0 ± 27.3 (137.0)	0.32
DBP (mmHg)	76.7 ± 11.7 (77.0)	75.6 ± 13.6 (76.0)	79.6 ± 8.3 (78.0)	0.63
BMI (kg/m^2^)	23.9 ± 3.1 (23.7)	24.3 ± 3.2 (24.0)	24.0 ± 4.4 (22.8)	0.45
WC (cm)	87.4 ± 9.1 (87.8)	89.5 ± 8.3 (90.0)	93.8 ± 9.5 (92.3)	0.08
HC (cm)	98.2 ± 5.8 (98.2)	97.2 ± 7.0 (97.5)	98.8 ± 7.8 (98.2)	0.65
WHR	0.89 ± 0.07 (0.88)^a^	0.92 ± 0.07 (0.92)^b^	0.95 ± 0.05 (0.95)^b^	**0.01**
CC (cm)	34.3 ± 2.8 (34.3)	34.8 ± 3.6 (34.5)	32.7 ± 2.0 (32.7)	0.11
Medical history (*n*, %)				
Heart disease	3 (5.4%)	11 (20.4%)	1 (10.0%)	0.06
Liver disease	2 (3.6%)	1 (1.9%)	0 (0.0%)	0.74
Kidney disease	1 (1.8%)	3 (5.6%)	1 (10.0%)	0.39
Hypertension	20 (35.7%)	22 (40.7%)	5 (50.0%)	0.66
Diabetes	9 (16.1%)	14 (25.9%)	3 (30.0%)	0.36
Hyperlipidemia	7 (12.5%)	11 (20.4%)	0 (0.0%)	0.20

*Note:* Bold values indicate statistically significant *p* values (*p* < 0.05).

Abbreviations: BMI, body mass index; CC, calf circumference; DBP, diastolic blood pressure; eGFR, estimated glomerular filtration rate; HC, hip circumference; SBP, systolic blood pressure; WHR, waist‐to‐hip ratio; WC, waist circumference.

^1^Mean ± standard deviation (median).

^a,b,c^Significant differences identified using post hoc tests when *p* < 0.05 in ANOVA or Kruskal–Wallis test.

Biochemical data of the participants are shown in Table 2. As expected, eGFR and creatinine levels differed significantly across groups, as the grouping was defined based on eGFR. Participants in the low and moderate eGFR groups had significantly higher HbA1_C_ levels compared with those in the high eGFR group (6.5 vs. 6.2 vs. 5.9%, *p* = 0.01). In addition, median levels of BUN (24.0 vs. 17.0 vs. 14.0 mg/dL, *p* < 0.01) and uric acid (5.6 vs. 5.3 vs. 4.6 mg/dL, *p* = 0.01) were significantly higher in the low and moderate eGFR groups. Moreover, the median homocysteine levels were also significantly elevated in the low and moderate groups than in the high eGFR group (16.9 vs. 11.9 vs. 9.3 μM, *p* < 0.01).

**TABLE 2:** Biochemical data of the participants according to the level of eGFR.

	**High eGFR (*N* = 56)**	**Moderate eGFR (*N* = 54)**	**Low eGFR (*N* = 10)**	**p** **value**

FBG (mg/dL)	106.6 ± 23.9 (99.0)^1^	109.7 ± 24.4 (102.5)	109.3 ± 30.0 (104.0)	0.61
HbA1_C_ (%)	6.1 ± 0.8 (5.9)^a^	6.4 ± 0.7 (6.2)^b^	6.5 ± 0.7 (6.5)^b^	**0.01**
TG (mg/dL)	114.0 ± 67.6 (103.0)	116.6 ± 56.4 (100.0)	111.1 ± 28.1 (104.5)	0.79
TC (mg/dL)	193.5 ± 34.8 (191.5)	191.6 ± 34.5 (187.0)	174.4 ± 37.7 (161.0)	0.28
LDL‐C (mg/dL)	113.2 ± 28.0 (113.0)	112.3 ± 31.1 (110.0)	100.0 ± 32.2 (89.5)	0.43
HDL‐C (mg/dL)	59.6 ± 17.9 (57.0)	58.3 ± 17.8 (55.0)	56.4 ± 10.2 (55.5)	0.76
TC/HDL‐C ratio	3.4 ± 0.9 (3.3)	3.5 ± 1.0 (3.3)	3.1 ± 0.7 (3.1)	0.45
AST (IU/L)	21.0 ± 4.2 (20.0)	21.8 ± 7.5 (20.0)	20.8 ± 3.4 (20.0)	0.93
ALT (IU/L)	18.6 ± 5.5 (17.0)	18.0 ± 6.9 (17.0)	17.0 ± 3.5 (17.0)	0.58
eGFR (mL/min/1.73 m^2^)	95.9 ± 3.4 (96.1)^a^	77.5 ± 8.5 (79.9)^b^	50.2 ± 9.9 (54.1)^c^	< 0.01**^∗^**
BUN (mg/dL)	14.9 ± 3.4 (14.0)^a^	17.9 ± 4.0 (17.0)^b^	25.0 ± 3.6 (24.0)^c^	**< 0.01**
Creatinine (mg/dL)	0.63 ± 0.10 (0.64)^a^	0.85 ± 0.15 (0.80)^b^	1.26 ± 0.33 (1.17)^c^	< 0.01**^∗^**
Uric acid (mg/dL)	4.8 ± 1.2 (4.6)^a^	5.3 ± 1.3 (5.3)^b^	5.7 ± 1.0 (5.6)^b^	**0.01**
Homocysteine (μM)	10.2 ± 3.2 (9.3)^a^	12.1 ± 3.1 (11.9)^b^	17.7 ± 4.8 (16.9)^c^	**< 0.01**
Folic acid (ng/mL)	10.4 ± 5.1 (8.7)	9.3 ± 4.2 (8.4)	9.1 ± 5.4 (8.1)	0.49
Vitamin B_12_ (pg/mL)	732.7 ± 403.7 (654.0)	845.0 ± 533.8 (646.0)	600.4 ± 283.5 (586.0)	0.44

*Note:* ALT, alanine aminotransferase; AST, aspartate aminotransferase; HbA1_C_, glycated hemoglobin; TG, triglyceride. *Note:* Bold values indicate statistically significant *p* values (*p* < 0.05).

Abbreviations: BUN, blood urea nitrogen; eGFR, estimated glomerular filtration rate; FBG, fasting blood glucose; HDL‐C, high‐density lipoprotein cholesterol; LDL‐C, low‐density lipoprotein cholesterol; TC, total cholesterol.

^1^Mean ± standard deviation (median).

^∗^ *p* values for eGFR and creatinine are presented for completeness, as group differences are inherent to the definition of eGFR categories.

^a,b,c^Significant differences identified using post hoc tests when *p* < 0.05 in ANOVA or Kruskal–Wallis test.

### 3.2. Regression Analysis of Renal Function Indicators, Folic Acid, Vitamin B_12_, and Homocysteine Levels Among Elderly Participants

**FIGURE 1:**
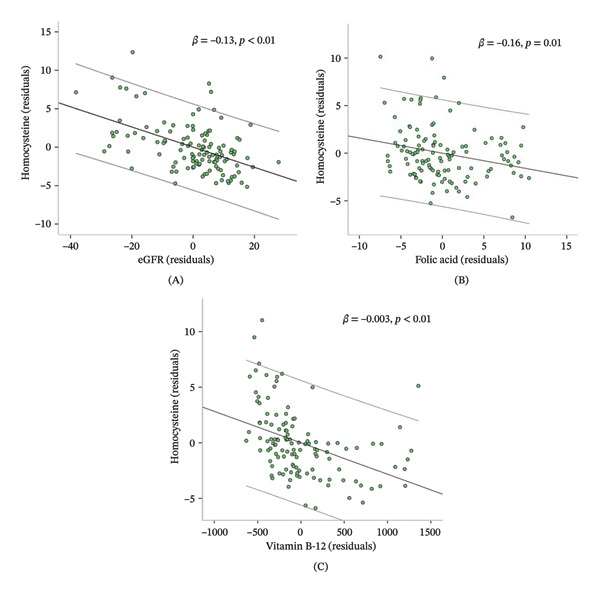
Partial regression plots illustrating the associations between renal function, nutritional factors, and homocysteine levels. (A) Association between eGFR and homocysteine. (B) Association between folic acid and homocysteine. (C) Association between vitamin B_12_ and homocysteine. Each plot shows the partial relationship between the predictor and homocysteine after adjustment for covariates (age, sex, heart disease, and the remaining predictors in the model). The axes represent residual values derived from the multivariable linear regression models. The central solid lines indicate the fitted regression lines, and the outer lines represent the 95% confidence intervals. The *β* coefficients and *p* values are derived from the corresponding multivariable regression analyses. eGFR, estimated glomerular filtration rate.

Given the increasing levels of homocysteine across decreasing eGFR categories, the associations between renal function indicators and homocysteine levels were examined using linear regression analysis, as shown in Table 3. The regression analysis showed that each 1‐unit decrease in eGFR was significantly associated with an increase of 0.13 μM in homocysteine levels (*β* = −0.13, *p* < 0.01). These associations remained significant after adjusting for age, gender, and heart disease. Folic acid (*β* = −0.29, *p* < 0.01) and vitamin B_12_ (*β* = −0.003, *p* < 0.01) were also significantly associated with homocysteine levels, and these associations remained significant after adjustment. Furthermore, in the fully adjusted model (Model 3) in which eGFR, folic acid, and vitamin B_12_ were simultaneously included, these associations remained statistically significant. To further illustrate the independent associations between renal function, nutritional factors, and homocysteine levels, partial regression plots were generated (Figure 1). These plots demonstrate that eGFR, folic acid, and vitamin B_12_ were inversely associated with homocysteine levels after adjustment for other covariates in the model. The linear trends observed in these plots are consistent with the results of the multivariable regression analysis. A sensitivity analysis further adjusting for WHR and HbA1_C_ yielded similar results, with the associations between eGFR, folic acid, vitamin B_12_, and homocysteine levels remaining statistically significant (Supporting Table S1).

**TABLE 3:** Multivariable linear regression analysis of the associations of renal function, folic acid, and vitamin B_12_ with homocysteine levels.

	**Homocysteine (μM)**					
	**Model 1**		**Model 2**		**Model 3**	
	***β* (95% CI)**	**p** **value**	***β* (95% CI)**	**p** **value**	***β* (95% CI)**	**p** **value**

*Each 1 unit*						
eGFR (mL/min/1.73 m^2^)	−0.13 (−0.17 to −0.09)	**< 0.01**	−0.13 (−0.18 to −0.08)	**< 0.01**	−0.13 (−0.17 to −0.09)	**< 0.01**
Folic acid (ng/mL)	−0.29 (−0.43 to −0.15)	**< 0.01**	−0.23 (−0.38 to −0.09)	**< 0.01**	−0.16 (−0.28 to −0.04)	**0.01**
Vitamin B_12_ (pg/mL)	−0.003 (−0.004 to −0.002)	**< 0.01**	−0.003 (−0.004 to −0.002)	**< 0.01**	−0.003 (−0.004 to −0.002)	**< 0.01**

*Note:* Model 1: univariable analysis; Model 2: adjusted for age, gender, and heart disease; Model 3: adjusted for age, sex, heart disease, eGFR, folic acid, and vitamin B_12_. *Note:* Bold values indicate statistically significant *p* values (*p* < 0.05).

Abbreviations: CI, confidence interval; eGFR, estimated glomerular filtration rate.

As a secondary analysis for clinical interpretation, we also evaluated the associations using a predefined categorical definition of hyperhomocysteinemia; these results are provided in Supporting Table S2. Each 1‐SD decrease in eGFR was significantly associated with an increased risk of hyperhomocysteinemia (OR: 2.63, *p* < 0.01), whereas no significant associations were observed for BUN and uric acid. With regard to nutritional factors, each 1‐SD increase in folic acid (OR: 0.54, *p* = 0.03) and vitamin B_12_ (OR: 0.42, *p* = 0.02) was associated with lower risk of hyperhomocysteinemia. These findings remained significant after adjustment for covariates.

### 3.3. Correlation Between Hyperhomocysteinemia and Metabolic Syndrome Factors Among Elderly Participants

**FIGURE 2:**
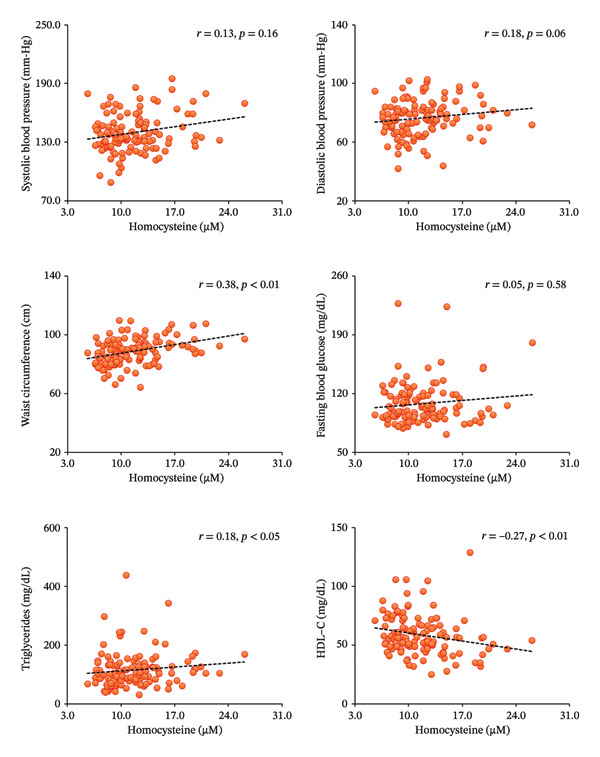
Correlations between homocysteine level and metabolic syndrome indicators in elderly subjects. HDL‐C, high‐density lipoprotein cholesterol.

The correlation between homocysteine levels and metabolic syndrome factors is shown in Figure 2. Homocysteine levels were significantly positively correlated with waist circumference (*r* = 0.38, *p* < 0.01) and TG levels (*r* = 0.18, *p* < 0.05) and negatively correlated with HDL‐C levels (*r* = −0.27, *p* < 0.01).

## 4. Discussion

Aging kidneys undergo structural changes, including a reduced number of functional glomeruli, tubular atrophy with interstitial fibrosis, and glomerulosclerosis, all of which contribute to a progressive decline in GFR with age [1]. In the present study, we found that participants in the low eGFR group were older and had higher WHR and serum uric acid levels than those in the high eGFR group (Tables 1 and 2). Obesity increases the metabolic and hemodynamic burden on the kidneys, potentially leading to compensatory glomerular hyperfiltration and tubular hypertrophy, which over time may accelerate renal function decline [15]. A reduction in eGFR may impair the kidneys’ ability to excrete uric acid, resulting in elevated serum uric acid levels [16]. Therefore, the combined effects of age‐related nephron loss, obesity, and hyperuricemia may promote nephron hypertrophy and contribute to the risk of CKD [17]. On average, GFR decreases by approximately 6.3 mL/min/1.73 m^2^ per decade [18]. However, it should be noted that eGFR‐based assessment has certain limitations, particularly in older adults, as age is incorporated into the estimation formula, which may influence its interpretation in clinical settings, particularly when used for diagnostic purposes [1]. The present study included community‐based older adults and did not specifically exclude those with renal disease, except in cases of severe or critical illness, in order to better reflect the actual conditions in this population. In this study, 8.3% of older adults had low eGFR, while 45.0% and 46.7% were classified as having moderate and high eGFR, respectively. According to the diagnostic criteria, CKD requires an eGFR < 60 mL/min/1.73 m^2^ persisting for at least 3 months [19]. Therefore, in the present study, eGFR values were not used to diagnose CKD, but rather to describe the renal function status of these older adults. In addition, the kidney plays an important role in glucose metabolism through gluconeogenesis in the proximal tubules [20]. Impaired renal function may be associated with alterations in glucose metabolism and regulation. A previous cross‐sectional study reported that more than half of patients with CKD had impaired fasting glucose [21]. Consistent with this observation, we found higher HbA1_C_ levels in the low eGFR group (Table 2). However, it is important to note that diabetes mellitus is a leading cause of reduced renal function [22], and the cross‐sectional design of this study does not allow determination of the direction of causality between renal function and glycemic status; rather, a bidirectional relationship likely exists. Both the low and moderate eGFR groups showed unfavorable profiles in metabolic parameters, including WHR, HbA1_C_, and renal markers, compared with the high eGFR group (Tables 1 and 2). These results suggest that eGFR may serve as a valuable indicator for monitoring metabolic and renal health in older adults.

Several cohort and cross‐sectional studies summarized in a systematic review and meta‐analysis demonstrated that individuals with elevated homocysteine levels had a higher risk of developing CKD compared with those with normal levels [23]. However, these studies did not specifically focus on older adults. We found that renal function and nutritional factors were significantly associated with homocysteine levels in community‐dwelling older adults (Table 3). Importantly, these associations remained statistically significant even after mutual adjustment for eGFR, folic acid, and vitamin B_12_, indicating complementary roles of renal function and nutritional factors in determining homocysteine concentrations. These findings extend previous literature by demonstrating that both physiological and nutritional factors contribute to homocysteine regulation through multiple pathways. In addition, we examined community‐dwelling older adults and compared homocysteine levels across high (≥ 90 mL/min/1.73 m^2^), moderate (< 90 mL/min/1.73 m^2^, ≥ 60 mL/min/1.73 m^2^), and low (< 60 mL/min/1.73 m^2^) eGFR groups. Homocysteine levels differed significantly among the three groups and showed a clear linear increasing trend. Compared with the high eGFR group, the median homocysteine level was 30.0% higher in the moderate eGFR group and 81.7% higher in the low eGFR group (Table 2). This finding implies that homocysteine levels rise markedly even at the moderate decline of kidney function. Although the effect size per unit change appears modest, cumulative declines in eGFR may be associated with clinically meaningful increases in homocysteine levels (Table 3). A possible mechanism is that impaired renal transsulfuration of homocysteine, together with decreased clearance through urinary excretion, contributes to this elevation [24]. Zhang et al. reported in a longitudinal study of older adults that during a 3.5‐year follow‐up period, eGFR declined from 90.8 to 77.2 mL/min/1.73 m^2^, accompanied by an increase in plasma homocysteine from 14.1 to 17.4 μM [25]. Similarly, a study in elderly individuals in Switzerland demonstrated a significant negative correlation between homocysteine and eGFR (*r* = −0.46, *p* < 0.01) and suggested that age‐related increases in homocysteine may partly reflect declining renal function [26]. Consistent with these findings, our study also observed higher homocysteine levels in participants with lower renal function. In addition, we further incorporated nutritional factors together with multiple renal function indicators to provide a more comprehensive evaluation of factors associated with homocysteine levels in older adults. Although other renal‐related markers, such as BUN, creatinine, and uric acid, showed associations with homocysteine levels in preliminary analyses, these variables were not included in the final regression model to avoid redundancy and to maintain a clear focus on eGFR as the primary indicator of renal function. However, when examining the risk of hyperhomocysteinemia, the associations with BUN and uric acid were no longer significant (Supporting Table S2). These findings highlight the stronger role of eGFR, compared with other renal function indicators, in its association with homocysteine. The definition of hyperhomocysteinemia was based on sex‐specific clinical reference values; however, such thresholds may vary across populations. Therefore, findings based on dichotomized outcomes should be interpreted with caution. Since homocysteine metabolism is largely regulated by the kidney but can also be modified by nutritional factors, it is well known that folic acid is converted to 5‐methyltetrahydrofolate, serving as a cofactor for methionine synthase together with vitamin B_12_ to remethylate homocysteine to methionine [27]. Accordingly, many trials have evaluated vitamin B supplementation and found that it can reduce homocysteine levels, particularly in stroke patients, as shown in a meta‐analysis of randomized controlled trials [28]. Although our study was not an intervention trial, we still observed significant associations of folic acid and vitamin B_12_ with homocysteine levels in older adults (Table 3). In Supporting Table S2, we further expressed results per 1 SD change in eGFR, folic acid, and vitamin B_12_ to provide supplementary clinical interpretation. The findings suggest that higher eGFR is strongly associated with lower homocysteine levels, followed by vitamin B_12_ and folic acid.

Several factors may contribute to hyperhomocysteinemia, including nutritional deficiencies of cofactors involved in homocysteine metabolism and kidney dysfunction, which can negatively affect the vascular system and promote atherosclerotic lesion progression [29]. In this study, we observed that elevated homocysteine levels and the risk of hyperhomocysteinemia were associated with impaired kidney function and possible nutritional deficiencies, as reflected in our findings (Table 3 and Supporting Table S2). In addition to these factors, our results also revealed that higher homocysteine concentrations were accompanied by unfavorable metabolic abnormalities, including increased waist circumference, elevated TG, and reduced HDL‐C (data not shown). Given the growing interest in life extension, previous studies have highlighted that homocysteine is linked to various age‐related disorders, such as neurodegeneration, cardiovascular complications, and osteoporosis [30]. These disorders are strongly associated with metabolic abnormalities, suggesting that homocysteine and related metabolic markers are particularly important indicators in older adults, not only because of their clinical relevance but also because they are relatively easy to monitor. In the present study, 20.8% of older adults were identified with hyperhomocysteinemia, and we also observed significant correlations between homocysteine levels and indicators of metabolic syndrome (Figure 2). As metabolic syndrome is known to increase the risk of falls, as well as all‐cause and cardiovascular mortality in older adults [31, 32], monitoring homocysteine levels may be particularly important in this population. Our findings highlighted potential risk factors of hyperhomocysteinemia and its adverse implications in older adults. These results suggest that attention to renal function and sufficient levels of folic acid and vitamin B_12_ may be important considerations in managing homocysteine concentrations.

We could not determine the causal relationship between renal function and homocysteine levels in older adults in this cross‐sectional study. Nevertheless, our findings demonstrated a clear association between poorer kidney function (reflected by lower eGFR and higher BUN, creatinine, and uric acid levels) and elevated homocysteine concentrations (Table 2). Since the decline in eGFR was strongly associated with hyperhomocysteinemia (Table 3), which may be accompanied by higher waist circumference and TG levels, greater attention should be paid to monitoring eGFR because even small changes may go unnoticed yet have significant implications in older adults. Long‐term, large‐scale studies are warranted to validate and extend our findings. These findings provide additional insight into the multifactorial regulation of homocysteine in older adults, highlighting the concurrent roles of renal function and nutritional status.

## 5. Conclusion

In this community‐based study of older adults, reduced eGFR was strongly associated with higher homocysteine levels, whereas higher folic acid and vitamin B_12_ were linked to lower homocysteine levels. Hyperhomocysteinemia also clustered with features of metabolic syndrome. These results highlight the interconnection between renal function, nutritional status, and metabolic health in aging. Early screening and attention to nutritional status may be important considerations in the management of these risks in aging.

## Author Contributions

Hsiu‐Lan Li and Po‐Sheng Chang conducted the study and recruited participants. Yao‐Mei Chuang and Po‐Jung Huang contributed to data collection and analysis. Po‐Sheng Chang conceived and designed the study and coordinated its implementation. Yao‐Mei Chuang and Po‐Sheng Chang drafted the manuscript.

## Funding

This work was supported by a grant from En Chu Kong Hospital, Taiwan (113‐COMP603A‐01).

## Disclosure

All authors have read and approved the final version of the manuscript.

## Ethics Statement

An ethical approval letter was obtained from the Institutional Review Board of En Chu Kong Hospital, Taiwan (IRB No. ECKIRB1130902). Written informed consent was sought from the study participants.

## Conflicts of Interest

The authors declare no conflicts of interest.

## Supporting Information

Additional supporting information can be found online in the Supporting Information section.

## Supporting information


**Supporting Information** The following supporting materials are available for this article. Supporting Table S1 presents the sensitivity analysis of the associations of eGFR, folic acid, and vitamin B_12_ with homocysteine levels after additional adjustment for waist‐to‐hip ratio and HbA1_C_. Supporting Table S2 presents the associations of renal function indicators and nutritional factors with hyperhomocysteinemia in the multivariable logistic regression analysis.

## Data Availability

The data supporting the findings of this study are available from the corresponding author upon reasonable request.
