# Supplementary material for: Estimated Glomerular Filtration Rate, Nutritional Factors, and Their Relationships With Homocysteine in Community‐Dwelling Older Adults
Source: J Nutr Metab. 2026 Jun 15;2026:7471621. doi: 10.1155/jnme/7471621 (PMC13266568; doi:10.1155/jnme/7471621)
Supplement: Supplementary file 1 — Supporting Information The following supporting materials are available for this article. Supporting Table S1 presents the sensitivity analysis of the associations of eGFR, folic acid, and vitamin B12 with homocysteine levels after additional adjustment for waist‐to‐hip ratio and HbA1C. Supporting Table S2 presents the associations of renal function indicators and nutritional factors with hyperhomocysteinemia in the multivariable logistic regression analysis. [file JNME-2026-7471621-s001.docx]

Supplementary Table S1: Sensitivity analysis of the associations of eGFR, folic acid, and vitamin B_12_ with homocysteine levels.

|  | Homocysteine (μM) | |
| --- | --- | --- |
| Each 1 unit increase | *β* (95% CI) | *p* value |
| eGFR (mL/min/1.73 m^2^) | -0.13 (-0.17 to -0.08) | **< 0.01** |
| Folic acid (ng/mL) | -0.16 (-0.28 to -0.04) | **0.01** |
| Vitamin B_12_ (pg/mL) | -0.003 (-0.004 to -0.002) | **< 0.01** |

Adjusted for age, sex, heart disease, waist-to-hip ratio (WHR), glycated hemoglobin (HbA1_C_), eGFR, folic acid, and vitamin B_12_. eGFR, estimated glomerular filtration rate; CI, confidence interval.

Supplementary Table S2: Factors associated with hyperhomocysteinemia in multivariable logistic regression analysis.

|  | Hyperhomocysteinemia | | | |
| --- | --- | --- | --- | --- |
|  | Model 1 | | Model 2 | |
|  | ORs (95% CI) | *p* value | ORs (95% CI) | *p* value |
| Each 1 SD | | | | |
| eGFR (mL/min/1.73 m^2^) | 0.38 (0.23 to 0.61) | **< 0.01** | 0.34 (0.19 to 0.60) | **< 0.01** |
| BUN (mg/dL) | 1.50 (0.97 to 2.32) | 0.07 | 1.40 (0.87 to 2.26) | 0.17 |
| Uric acid (mg/dL) | 1.34 (0.87 to 2.06) | 0.19 | 1.39 (0.89 to 2.17) | 0.15 |
| Folic acid (ng/mL) | 0.54 (0.30 to 0.95) | **0.03** | 0.52 (0.29 to 0.95) | **0.03** |
| Vitamin B_12_ (pg/mL) | 0.42 (0.20 to 0.85) | **0.02** | 0.43 (0.21 to 0.87) | **0.02** |

Model 1: Univariable analysis; Model 2: Adjusted for age, gender, and heart disease. BUN, blood urea nitrogen; CI, confidence interval; eGFR, estimated glomerular filtration rate; ORs, odds ratios; SD, standard deviation.
